# Cadmium-Induced Neuroendocrine Alterations: Gene Expression of the Kisspeptin–GnRH Axis and Delayed Puberty in Male Rats

**DOI:** 10.3390/toxics14030270

**Published:** 2026-03-22

**Authors:** Marcela Arteaga-Silva, Eduardo Miguel Cornejo de la Concha, Daniel Adrian Landero-Huerta, Sergio Montes, Julio César Rojas-Castañeda, Rosa María Vigueras-Villaseñor, Joel Hernández-Rodríguez, Sergio Marín de Jesús, Sonia Guadalupe Pérez-Aguirre, Rocío Trilce López-Ruíz, Isabel Arrieta-Cruz

**Affiliations:** 1 Laboratorio de Neuroendocrinología Reproductiva, Departamento de Biología de la Reproducción, División de Ciencias Biológicas y de la Salud, Universidad Autónoma Metropolitana-Iztapalapa, Av. Ferrocarril San Rafael Atlixco No. 186, Col. Leyes de Reforma 1ª, Sección, Alcaldía Iztapalapa, Ciudad de México 09340, Mexico; uamarinsergio@gmail.com (S.M.d.J.); soniagaguirre@gmail.com (S.G.P.-A.); trilceroh@gmail.com (R.T.L.-R.); 2 Posgrado en Biología de la Reproducción Animal, División de Ciencias Biológicas y de la Salud, Universidad Autónoma Metropolitana-Iztapalapa, Av. Ferrocarril San Rafael Atlixco No. 186, Col. Leyes de Reforma 1ª, Sección, Alcaldía Iztapalapa, Ciudad de México 09340, Mexico; lalocornejo33@gmail.com; 3 Laboratorio de Biología de la Reproducción, Instituto Nacional de Pediatría, Ciudad de México 04530, Mexico; qdanielfadrianb@hotmail.com (D.A.L.-H.); rocajc1@yahoo.com.mx (J.C.R.-C.); rmvigueras@yahoo.com.mx (R.M.V.-V.); 4 Unidad Académica Multidisciplinaria Reynosa-Aztlán, Universidad Autónoma de Tamaulipas, Lago de Chapala y Calle 16, Aztlán, Reynosa 88740, Mexico; sergio.montes@uat.edu.mx; 5 Cuerpo Académico de Investigación en Salud de la Licenciatura en Quiropráctica (CA-UNEVE-01), Universidad Estatal del Valle de Ecatepec, Ecatepec de Morelos 55210, Mexico; joelhr19@hotmail.com; 6 Departamento de Investigación Básica, Instituto Nacional de Geriatría, Secretaría de Salud, Ciudad de México 10200, Mexico; arrieta777@mail.com

**Keywords:** puberty, kisspeptin, *Kiss1*, *Kiss1r*, *Gnrh1*, cadmium

## Abstract

Puberty is a neuroendocrine process required for sexual maturity; it is regulated by the hypothalamic–hypophysis–gonadal (HHG) axis. Kisspeptin (KISS1) plays a vital role in activating this axis by stimulating the secretion of gonadotropin-releasing hormone (GnRH). Cadmium (Cd) exposure disrupts KISS1 signaling in female rodents; its effects on hypothalamic gene expression during male puberty remain poorly understood. This study investigated the effects of Cd exposure on hypothalamic *Kiss1*, *Kiss1r*, and *Gnrh1* expression, preputial separation (PS) as a marker of pubertal onset, testosterone levels, Cd concentration, and total antioxidant capacity (TAC) in the serum and hypothalamus of pubertal male Wistar rats. Animals received once a week intraperitoneal injection of CdCl_2_ (1 mg/Kg body weight/100 µL) or saline (100 µL) and were euthanized on postnatal day (PND) 35 or 49. Cd exposure reduced serum testosterone levels and TAC. Also, pubertal onset was delayed. At PND 35, Cd decreased hypothalamic *Kiss1* expression, whereas at PND 49, it reduced *Kiss1r* and *Gnrh1* expression. These results suggest that Cd alters hypothalamic gene expression, which may contribute to delayed puberty and impaired sexual maturity. Our findings suggest the vulnerability of puberty to exposure to Cd, acting as an endocrine disruptor and neurotoxicant, with alterations for male reproductive maturity.

## 1. Introduction

Puberty is a critical stage of development that marks the reaching of sexual maturity, which culminates in the acquisition of reproductive capacity. In mammals, including humans, this process is controlled by neuroendocrine changes that are highly regulated by the kisspeptin (KISS1), which involves the activation of the hypothalamic-hypophysis-gonadal (HHG) axis [1,2,3]. During this period, the presence of neurons co-expressing KISS1, neurokinin B, and dynorphin A (KNDy), essential components of the GnRH pulse generator, has been described in the arcuate nucleus (ARC) of the hypothalamus, where neurokinin B exerts a stimulating effect, while dynorphin A acts as an inhibitory modulator of the synchronized activity of KNDy neurons through paracrine mechanisms [3,4]. KISS1 is encoded by the *Kiss1* gene and expressed in KISS1 neurons [4]. KISS1 acts in the median eminence (ME) through its receptor (Kiss1r), and allows the release of GnRH [5,6]. Therefore, its secretion is crucial for the increase in gonadotropins, including luteinizing hormone (LH) and follicle-stimulating hormone (FSH), during puberty, which, in turn, promotes testicular steroidogenesis and male sexual maturity [1,7]. It has been shown that pubertal development is associated with increased activity of the GnRH neuronal system and increased concentrations of gonadal steroids [3,8,9,10] compared to adulthood, where there is significant GnRH secretion and elevated concentrations of LH and testosterone; this occurs even though the distribution and projections to the ME of the GnRH neural network are similar in both the pubertal and adult stages [7,11]. In addition, the distal projections of GnRH neurons receive synaptic information and respond to KISS1 [12]. Thus, puberty is the result of the interaction among neural signals, hormones, and environmental factors [4,13,14]. In male rats, the functional maturity of the HHG axis and reproductive system occurs mainly during the postnatal period. Thus, four well-defined stages have been proposed: neonatal (birth to postnatal day [PND] 7), infantile (PND 8–20), juvenile (PND 21–32), and peripuberal (PND 33–55/60), each characterized by specific cellular, hormonal, and morphological events [15,16,17]. At birth (PDN 1), LH receptors and testosterone production are detectable until maturity is reached. Between PDN 26–35, GnRH levels reach high levels; during the juvenile period, the hypothalamus and pituitary gland become progressively less sensitive to negative feedback from testosterone, allowing for an increase in FSH, LH, and T, which coincides with the maturation of GnRH neuronal pulsatility at PND 30–40 [17,18]. From a morphological perspective, in male rats, puberty is commonly assessed by preputial separation (PS), a morphological marker that depends on sustained androgen production for its occurrence [19].

The study of puberty has also been approached at the level of gene expression for the transcripts *Kiss1*, *Gnrh1*, *Kiss1r*, and *Gnrhr* [4,6,10,20], as well as for other transcripts involved in the HHG axis, such as *Lhb* and *Fshb* [10,21], and even transcripts involved in steroidogenesis in the gonad, such as *Star* [22], among others. Puberty is a stage of development regulated at the epigenetic level [4,6,10,20]. Since the onset of puberty requires finely controlled regulation, this period represents a stage of special susceptibility to epigenetically regulated exposures [4,6,10,20]. Endocrine disruptors are defined as exogenous substances that can interfere with the synthesis, release, transport, action, or elimination of hormones necessary for homeostasis and normal development of the organism [23]. In this context, heavy metals are recognized as highly relevant pollutants due to their widespread presence in the environment, their high persistence, and their ability to bioaccumulate in organisms over time [23,24,25]. Among these, cadmium (Cd) stands out as a non-essential metal classified as an endocrine disruptor and a carcinogen, capable of altering various neuroendocrine processes, particularly those related to male reproduction [26,27,28,29,30,31]. Recent studies suggest that the disruptive action of Cd can occur at various stages of development, affecting key events of puberty, such as the establishment of the pulsatile pattern of GnRH and testosterone secretion [31,32]. Cd is known to reduce testosterone synthesis by inhibiting steroidogenic enzymes, oxidative stress, and mitochondrial damage [33,34,35]. These alterations can result in delayed PS, testicular hypotrophy, and redox alterations [35,36]. Furthermore, it has been demonstrated that Cd-induced testicular damage interferes with the feedback provided to brain regions responsible for reproductive control, such as the hypothalamus [26,37]. This is because Cd can cross the blood–brain barrier (BBB) and induce neurotoxicity [38,39]. On the other hand, exposure to Cd in adult female rats is known to alter the transcriptome of neurological diseases, in addition to inducing histopathological changes in the ARC [40]. Specifically, a reduction in *GnRH* expression and altered HHG activity was observed in prepubertal female rats [41]. However, the effects of Cd on the expression of *Kiss1* and *GnRh1* in male rats are unknown. The objective of this study was to analyze the Cd exposure on hypothalamic *Kiss1*, *Kiss1r*, and *Gnrh1* expression, PS as a marker of pubertal onset, testosterone levels, blood, and the hypothalamus Cd concentration, and total antioxidant capacity (TAC) in serum and hypothalamus of pubertal male Wistar rats.

## 2. Materials and Methods

### 2.1. Animals and Treatments

Twelve gestating Wistar rats were housed individually at the Bioterio of the Universidad Autónoma Metropolitana-Iztapalapa. They were maintained under a 12 h light–dark cycle, with the lights turned off at 8:00 a.m. The temperature was kept at 24 ± 1 °C. The rats had ad libitum access to rat chow (Harlan Laboratories, Indianapolis, IN) and water. All experimental procedures and the handling of these animals followed the National Institutes of Health (NIH, 2011) and Mexico’s Official Norm (NOM-062-ZOO-1999, reviewed in 2001) for the care and handling of laboratory animals, and in conjunction with the institutional ethics committee (Conducción ética de la investigación, la docencia y la difusión en la División de Ciencias Biológicas y de la Salud de la UAM-Iztapalapa). Experiments were conducted with the minimum number of animals necessary and without causing suffering.

On PND 1, the pups from these dams were sexed, and only males were included in this study. Their initial body weight was recorded (6.727 ± 0.140 g) to ensure homogeneity among the experimental groups. A total of 68 pups were randomly assigned to the experimental groups. The experimental animals were treated as follows: two groups were injected once per week with 1 mg/Kg body weight/100 µL of cadmium chloride (CdCl_2_) (Sigma-Aldrich, St. Louis, MO, USA), diluted in saline solution. One group was injected intraperitoneally (IP) until PND 35, and the other until PND 49. According to the developmental events occurring at seven-day intervals during pubertal development described by Picut et al. [16], this administration schedule was selected to evaluate Cd-induced toxicity. The once-weekly dosing of 1 mg/Kg body weight allowed the assessment of cumulative Cd exposure, which more closely mimicked environmental or occupational exposure conditions [42,43,44]. This administration scheme has also been reported to reduce acute handling stress in experimental rats [45,46,47].

After birth, the pups were placed under the care of a surrogate dam until weaning. Animals were euthanized on PND 35 and PND 49 for subsequent biochemical and molecular analyses, including serum testosterone quantification and Cd determination in blood and hypothalamus (*n* = 6 per group), TAC assay (*n* = 6 per group), and RNA isolation followed by real-time quantitative PCR analysis (*n* = 5 per group).

### 2.2. Experimental Procedure

Four experimental groups of Wistar rats were established. Two groups were treated with CdCl_2_ at a dose of 1 mg/Kg body weight/100 µL administered by IP injection once per week from PND 1 until PND 35 or PND 49. The remaining two control (Ctrl) groups received saline solution following the same administration schedule.

**Figure 1:**
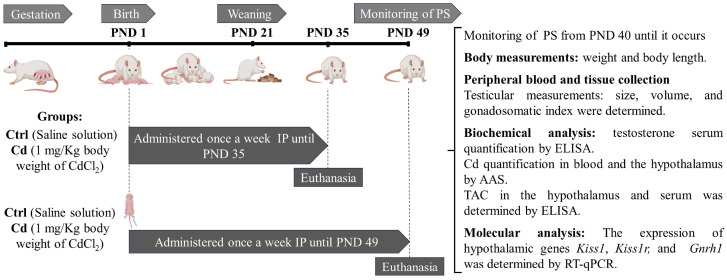
Four experimental groups of Wistar rats were established. Two Ctrl groups received saline solution from PND 1 to PND 35 or PND 49. The other two groups received Cd (1 mg/Kg body weight/100 µL) from PND 1 to PND 35 or PND 49. All administrations were performed once per week via IP injection. PS was monitored daily from PND 40 to PND 49 until it occurred. At the end of the treatment period, body weight was recorded, animals were euthanized, blood samples were collected, and the testes and hypothalamus were dissected. Testicular weight, length, and volume were measured, and the gonadosomatic index was calculated. Serum testosterone levels were quantified using ELISA. Cd concentration in blood and the hypothalamus was determined by AAS. TAC in serum and hypothalamic tissue was quantified by ELISA. Additionally, hypothalamic gene expression of *Kiss1*, *Kiss1r*, and *Gnrh1* was analyzed by RT-qPCR. Abbreviations: control (Ctrl), postnatal days (PND), intraperitoneal (IP), preputial separation (PS), enzyme-linked immunosorbent assay (ELISA), atomic absorption spectrophotometry (AAS), Total antioxidant capacity (TAC), and real-time quantitative polymerase chain reaction (RT-qPCR).

Animals were euthanized by decapitation at PND 35 or PND 49. The testes were dissected, weighed, and their length was measured by a single observer. The gonadosomatic index was calculated by dividing the weight of each testis by the body weight and multiplying the result by 100 (testis weight/body weight × 100). Peripheral blood samples were collected in metal-free Vacutainer tubes and subsequently analyzed to determine Cd concentration using atomic absorption spectrophotometry (AAS). Serum testosterone levels were quantified using an enzyme-linked immunosorbent assay (ELISA). In addition, total antioxidant capacity (TAC) was measured in both serum and hypothalamic tissue. Finally, the expression levels of the *Kiss1*, *Kiss1r*, and *Gnrh1* genes in the hypothalamus were evaluated by reverse transcription quantitative polymerase chain reaction (RT-qPCR) (Figure 1).

### 2.3. Morphological Analysis

#### Puberty Marker

The rats’ pubertal stage was assessed by monitoring PS in the groups of PND 49, by a single observer, from PND 40 to PND 49. PS was recorded by placing the rat in a supine position and retracting the prepuce. The prepuce was manipulated with the fingers without forcing or applying pressure. The PS was considered when the glans penis was completely separated.

### 2.4. Tissue Collection

At the end of each treatment period, animals were euthanized by decapitation.

#### 2.4.1. Testes Tissue Collection

The testes of the animals in the four groups were dissected, weighed, and their length measured; the GSI was calculated.

#### 2.4.2. Hypothalamic Tissue Collection

Hypothalamic tissues from the four experimental groups were removed and dissected under aseptic conditions at 4 °C. The samples were immediately stored at −80 °C until further analysis.

### 2.5. Biochemical Analysis

#### 2.5.1. Testosterone Serum Quantification

Blood samples were obtained by decapitating each rat between 13:00 and 14:00 to avoid circadian variations and were collected in tubes with serum separator stoppers (BD Vacutainer SST, Mexico City, Mexico). Serum was collected through centrifugation for 15 min at 3000 rpm. Free testosterone was determined by ELISA. Each sample was analyzed in duplicate using a commercial kit (Testosterone ELISA catalog number EIA-1559, DRG, Springfield, NJ, USA). Calibration curves were generated for each analysis utilizing reference standards (0, 0.2, 0.5, 1.0, 2.0, 6.0, and 16.0 ng/mL), with an R^2^ value of 0.98. The kit was read with a UV-Vis spectrophotometer (PerkinElmer Lambda 40, Norwalk, CT, USA) at a wavelength of 450 nm. Serum testosterone levels were quantified in ng/mL.

#### 2.5.2. Quantification of Cd in Blood and the Hypothalamus

The Cd content in blood and the hypothalamus was determined according to the Sharma et al. protocol [48], using an atomic absorption spectrophotometer (AAS) (PerkinElmer PinAAcle Model AS900; Norwalk, CT, USA) with a graphite furnace (THGA) and sampler (AS900, PerkinElmer), with the furnace set to a wavelength of 228.8 nm. For each analysis, calibration curves were constructed using aqueous Cd reference standards (0.5, 1.0, 2.0, 4.0, and 6.0 µg/L), with an R^2^ value of 0.98. To optimize and validate the measurements, Cd quality control measurements were performed at the beginning and end of each analysis using a previously digested bovine liver standard solution (NIST 1577b, Gaithersburg, MD, USA) [49]. Concentrations of Cd were expressed as µg/mL in blood samples and µg/g in tissue samples.

#### 2.5.3. Total Antioxidant Capacity Assay

The hypothalamus was homogenized in cold PBS buffer and subsequently centrifuged at 10,000 rpm for 10 min at 4 °C. Serum was obtained by centrifugation at 3000 rpm for 15 min at room temperature. Aliquots of 20 µL of serum and of the supernatant from the homogenized tissues were taken to determine the TAC. Each sample was quantified in duplicate using a commercial kit (Cell Biolabs, OxiSelect™ TAC catalog number STA-360; Lot 3602506, San Diego, CA, USA). The kit was read using a UV-Vis spectrophotometer (PerkinElmer Lambda 40, Norwalk, CT, USA) at a wavelength of 490 nm. Uric acid equivalent ‘μM Copper Reducing Equivalents’ were determined for each sample according to the kit. The fundamental assay principle of Cell Biolabs’ OxiSelect™ is that TAC measures the total antioxidant capacity within a sample, which is based on the reduction of copper (II) to copper (I) by antioxidants, such as uric acid. Following this, the copper ion (I) reacts with a chromogenic coupling reagent to produce a color. Samples are compared to a known concentration of uric acid standard within a 96-well microtiter plate format. Calibration curves were constructed using uric acid concentration reference standards (0.0, 0.0039, 0.0078, 0.3125, 0.0625, 0.125, 0.25, 0.5, and 0.1 mM), with an R^2^ value of 0.97. Samples and standards are diluted with a reaction reagent, and upon the addition of copper, the reaction proceeds for a few minutes. The reaction is stopped and read at 490 nm. Antioxidant capacity is determined by comparison with the uric acid standards.

### 2.6. Molecular Analysis

#### RNA Isolation and Real-Time Quantitative PCR Analysis

Hypothalamic tissue was dissected and stored at −80 °C until further processing. Total RNA was extracted from these samples according to the manufacturer’s instructions using the QIAGEN^®^ RNeasy Mini Kit (Hilden, Germany). Reverse transcription of *Kiss1*, *Kiss1r*, and *Gnrh1* transcripts was performed using the TaqMan^®^ Reverse Transcription Reagents commercial kit (Applied Biosystems, Waltham, MA, USA). For each reaction, 100 ng of total RNA was reverse transcribed using looped RT primers (Applied Biosystems, Waltham, MA, USA) according to the manufacturer’s protocol.

qPCR assays were performed in triplicate using an Applied Biosystems StepOne™ thermocycler (Foster City, CA, USA). Each reaction contained 2 µL of cDNA, TaqMan^®^ Gene Expression probes specific to each transcript (Applied Biosystems, Waltham, MA, USA; see Table 1), and TaqMan^®^ Universal PCR Master Mix (Applied Biosystems, Waltham, MA, USA). The relative gene expression levels were calculated using the 2^−ΔΔ^Ct comparative method, with *Gapdh* used as the endogenous reference gene for normalization. RNase-free H_2_O was included as the negative control in all reactions.

The technical characteristics of the TaqMan^®^ Gene Expression assays used for *Kiss1*, *Kiss1r*, *Gnrh1*, and *Gapdh* are shown in Table 1.

### 2.7. Statistical Analysis

All parameters were expressed as the mean ± SEM. The normal distribution of the data within each group was verified using the Shapiro–Wilk test. Data obtained from the PS, body weight and size, testicular weight and volume, gonadosomatic index, testosterone levels, Cd concentration and TAC, and relative expression were analyzed using a Student’s *t*-test (*p* < 0.05). All analyses were performed using GraphPad version 8.0 (La Jolla, CA, USA).

## 3. Results

### 3.1. Effects of Cd Exposure on Body and Testicular Morphometric Parameters

No physical signs indicative of systemic toxicity were observed in any of the groups throughout the treatment period. Regarding PS, the Ctrl group reached the puberty index at PND 41.7 ± 0.494, while the Cd group showed a significant delay of 6 days (47.0 ± 0.516). Table 2 shows the body weight for each treatment group at the two ages evaluated. A significant difference in final body weight was observed between the Cd and Ctrl groups at PND 35 (*p* = 0.026) and PND 49 (*p* = 0.043), with Cd exposure resulting in decreased body weight. Significant differences in body length were also observed between the Cd group and the Ctrl group at PND 35 (*p* = 0.0005) and PND 49 (*p* = 0.0081), with Cd-treated rats showing reduced body length at both ages. Regarding testicular weight, no significant difference was observed between the groups at PND 35 (*p* = 0.063); however, a significant difference between the Cd and Ctrl groups was observed at PND 49 (*p* < 0.0001), with Cd-exposed rats presenting reduced testicular weight. When testicular volume was compared between the evaluated groups, no significant differences were observed at PND 35 (*p* = 0.37). However, a significant difference was observed at PND 49 (*p* < 0.0001) with the Cd group presenting a smaller testicular volume than the Ctrl group.

Regarding the gonadosomatic index, no significant differences were observed between the groups at PND 35 (*p* = 0.24). However, a significant difference was observed at PND 49, where Cd exposure resulted in a decrease in the gonadosomatic index in the Cd group compared with the Ctrl group (*p* < 0.0001).

### 3.2. Biochemical Evaluation 

Table 3 shows the effects of Cd on serum testosterone levels, its bioaccumulation in hypothalamic tissue, and its presence in blood, as well as its impact on the TAC of hypothalamic tissue and serum. Regarding testosterone levels, a significant difference was observed between the evaluated groups at PND 35, with the Cd group showing lower testosterone levels than the Ctrl group (*p* = 0.0067). This decrease remained significant at PND 49 (*p* < 0.0001). Regarding Cd concentration in hypothalamic tissue, a notable difference was observed at PND 35, with the Cd group showing clear accumulation of the metal compared with the Ctrl group (*p* = 0.0004). The same pattern of Cd accumulation in hypothalamic tissue was observed at PND 49, with significantly higher Cd levels in the Cd group compared with the Ctrl group (*p* < 0.0001). Blood Cd concentrations were significantly higher in the Cd group than in Ctrl group at PND 35 (*p* < 0.0001) and PND 49 (*p* < 0.0001). Significant differences in TAC concentration in hypothalamic tissue were observed between the groups. In both cases, the Cd-exposed group exhibited a reduced TAC concentrations compared with the Ctrl group at PND 35 (*p* < 0.001) and PND 49 (*p* < 0.040). Serum TAC concentrations also showed significant differences when the data from each age group were analyzed, indicating that Cd exposure induced a significant decrease in TAC at PND 35 (*p* < 0.006) and PND 49 (*p* < 0.001) compared with the corresponding Ctrl group.

### 3.3. Relative Expression of Kiss1, Kiss1r and Gnrh1 in Hypothalamus

**Figure 2:**
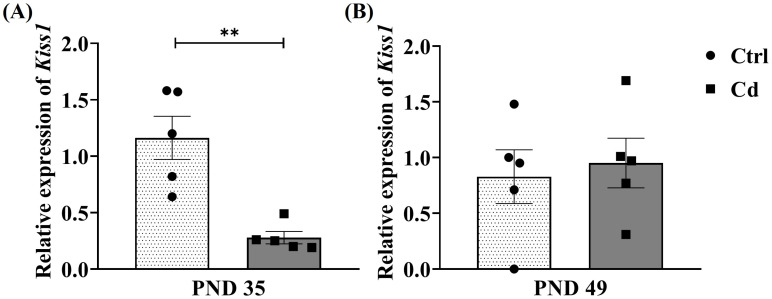
Cd effect on the hypothalamic relative expression of *Kiss1*. The graph shows the changes in relative expression of *Kiss1* between the Cd and Ctrl groups at (**A**) PND 35 and (**B**) PND 49. Data expressed as means ± SEM for all analyzed groups (*n* = 5 per group). ** *p* < 0.01.

The Cd effects on the relative expression of *Kiss1* in rats evaluated at both ages are shown in Figure 2. At PND 35, a decrease in the relative expression of this transcript was observed in rats from the Cd group compared with the Ctrl group, resulting in a significant difference (*p* = 0.0022). However, at PND 49, although a variation in expression was observed between the Cd and Ctrl groups, no significant differences were obtained (*p* = 0.72).

**Figure 3:**
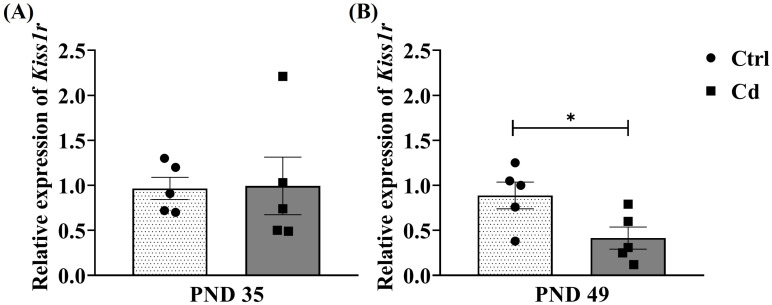
Cd effect on the hypothalamic relative expression of *Kiss1r*. The graph shows the changes in the relative expression for *Kiss1r* between the Cd and Ctrl groups at (**A**) PND 35 and (**B**) PND 49. Data expressed as means ± SEM for all analyzed groups (*n* = 5 per group). * *p* < 0.05.

Figure 3 shows the effect of Cd on the relative expression of *Kiss1r* at PND 35 and PND 49. At PND 35, no significant differences were observed in the Cd group compared with the Ctrl group (*p* = 0.9368). However, at PND 49, a significant decrease in relative expression was observed in the Cd group compared with the Ctrl group (*p* = 0.039).

**Figure 4:**
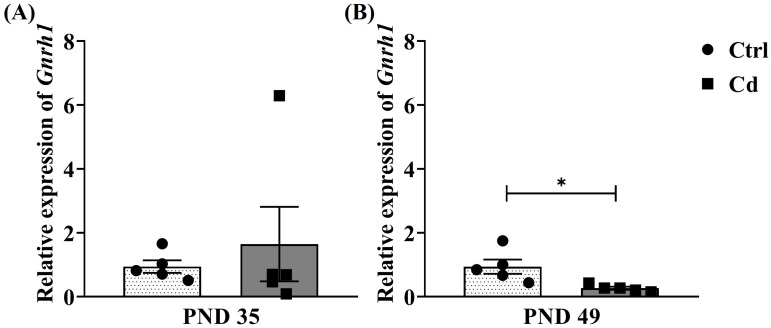
Cd effect on hypothalamic relative expression of *Gnrh1*. The graph shows the changes in the relative expression for *Gnrh1* between the Cd and Ctrl groups at (**A**) PND 35 and (**B**) PND 49. Data expressed as means ± SEM for all analyzed groups (*n* = 5 per group). * *p* < 0.05.

Finally, Figure 4 shows the relative expression of *Gnrh1*. At PND 35, rats in the Cd group showed an increase in the relative expression of this transcript. However, no significant differences were found when compared with the Ctrl group (*p* = 0.57). In contrast, when the data from both groups were evaluated at PND 49, a significant difference was observed; with the Cd group exhibiting decreased relative expression compared with the Ctrl group (*p* = 0.018).

## 4. Discussion

This study demonstrates that postnatal exposure to Cd (1 mg/Kg body weight/once per week, IP) until puberty in male Wistar rats induces a delay in PS and neuroendocrine disruption associated with systemic and hypothalamic oxidative stress. Cd is widely present in the environment and is a byproduct of anthropogenic activities. Thus, exposure to Cd is continuous in both humans and animals, even at low concentrations [50,51,52,53]. Its toxicity affects multiple organ systems, including the HHG endocrine axis [30,32,54]. In particular, Cd has been characterized as an endocrine disruptor capable of interfering with hormonal homeostasis and neuroendocrine signaling that regulates reproductive functions [26,31,32,34,55]. Our results showed that Cd exposure caused a delay in PS. These findings suggest alterations in markers of sexual maturation in rats, similar to the results reported by Interdonato et al. [56]. Adolescents living in the Milazzo-Valle del Mela area of Sicily, Italy, have been reported to exhibit higher concentrations of Cd, delayed puberty onset, smaller testicular volume, and lower testosterone levels. A more recent study reported that peripubertal Cd exposure in children aged eight to fourteen years in Mexico City, Mexico, resulted in decreased testosterone levels [57]. In terms of body weight, animals exposed to Cd showed modest weight loss compared to the Ctrl group on DPN 35, an effect previously reported [58,59]. However, a clear decrease in body weight was observed in the animals at PND 49. This may be due to the phenomenon reported by Kawakami et al. [60] in adult rats, whereby Cd induces adipocyte apoptosis in white adipose tissue. This results in a significant reduction in white adipose tissue weight, as well as in the weight of other tissues and, consequently, body weight. In this same group, there was also a decrease in animal length. Although few studies analyze the length of Cd-exposed animals, the results obtained are consistent with those reported by Rodríguez and Mandalunis [61]. They mention that bone is a dynamic structure that undergoes constant remodeling throughout life, requiring precise coordination of bone matrix synthesis and mineralization by osteoclasts and osteoblasts. Therefore, any factor that interferes with the function of these cells will cause alterations in their processes. Cd can interfere with bone remodeling during growth by stimulating osteoclastogenesis and inhibiting osteoblast differentiation. This results in a decrease in bone volume and a decrease in animal length, as demonstrated in the femur of rats [62] administered with the same dose used in our study. However, further studies are needed to analyze the effects of Cd on the reduction in body length and its mechanisms. Regarding testicular volume, the decrease in this parameter was more evident by PND 49, as was the gonadosomatic index. Morphometric indices, such as the gonadosomatic index, standardize organ weight in relation to body weight. This demonstrates specific effects on the testicle regardless of the animal’s length. This is important in toxicological research when body weight alterations occur [63]. The decrease in the gonadosomatic index in our results is indicative of the toxic effect of Cd on the testes by inducing a proportional reduction in body weight, as reported by Ravkash et al. [63] and in other studies. These studies have reported Cd accumulation in the testes after postnatal exposure, which reduces testicular weight [35,53] and the gonadosomatic index [36,64]. Testosterone, on the other hand, is an indicator of the onset of puberty and HHG axis activity [26,58,65]. Our results indicate that Cd reduces serum testosterone levels. The direct interaction of Cd with the testis has been proposed to manifest as histological, morphological, and structural changes in various cell types, including germ cells, Sertoli cells, Leydig cells, and spermatogenic cells [53,66,67,68]. Cd has also been shown to alter hormone synthesis and regulation, even at low doses [30,69], particularly the synthesis of steroidogenic enzymes such as 3β- and 17β-hydroxysteroid dehydrogenase, which are crucial for androgen metabolism [70,71]. Exposure to Cd at doses similar to those used in this study can cause significant imbalances in testosterone synthesis due to deficiencies or reductions in the activity of these enzymes [67,71,72,73]. While this study did not measure GnRH, FSH, or LH levels, reductions in these hormones have been reported in response to Cd exposure [34,74].

Regarding the bioaccumulation of Cd in blood and hypothalamic tissue, this study observed an increase in concentration in both age groups. Cd accumulates in the body and is excreted in very low percentages [75], with a very long half-life. After exposure and absorption, Cd enters the bloodstream, binds to erythrocyte membranes, blood albumin, and metallothioneins, and is distributed throughout the body. There, it accumulates and is stored in various organs, causing adverse effects [75,76]. These findings suggest that Cd reaches the central nervous system during puberty. Cd easily crosses the immature BBB of young animals [40,77], and can affect different brain structures [78,79], including the hypothalamus [37,40,80,81]. Therefore, Cd bioaccumulation in the hypothalamus may lead to neurotoxicity and affect neuronal processes necessary for pubertal development, making it a sensitive window to endocrine disruptors such as Cd. Additionally, a reduction in TAC was observed in both serum and the hypothalamus [37]. This finding supports the hypothesis that Cd generates systemic oxidative stress in both tissues [82,83,84,85] by increasing reactive oxygen species and inducing cell damage, membrane alterations, and endocrine signal dysfunction [83,86,87]. Furthermore, Cd decreases the activity of the redox system [35,36] and could therefore alter the activation and consolidation of the kisspeptin-GnRH network during puberty.

Among the adverse effects of Cd, it has been reported that gene dysregulation may occur due to direct effects such as cell injury, oxidative stress, and alterations in DNA, including methylation. The main toxic mechanisms through which Cd can interfere with calcium (Ca^2+^) signaling through ionic mimicry [88]. It can enter neurons through voltage-activated Ca^2+^ channels [89] and subsequently exert molecular mimicry due to its high affinity for the sulfhydryl (-SH) groups present in glutathione (GSH) and the cysteine residues of proteins [88]. This mechanism reduces the cell’s capacity to neutralize organic hydroperoxides, thereby increasing the susceptibility of proteins to structural changes and loss of function [90]. This causes genotoxic effects due to oxidative damage. Cd can activate Ca^2+^-dependent enzymes such as calpains and endonucleases, which degrade structural and nuclear proteins. This results in DNA fragmentation, cytoskeletal degradation, and, ultimately, cell death via apoptosis [88]. Additionally, Cd inhibits DNA repair [91,92], resulting in DNA methylation and dysregulation of gene expression [93].

Furthermore, Cd can induce long-term changes in gene expression through epigenetic alterations, such as DNA hypomethylation, which is associated with genomic instability and an increased mutation rate [94]. Cd can also cause localized hypermethylation of specific suppressor genes to result in transcriptional silencing [94]. Moreover, Cd can modulate acetylation and methylation, which are post-translational histone modifications, thereby altering chromatin accessibility and gene expression [95,96]. Cd can also affect the expression of microRNAs, which contribute to alterations in cell cycle regulation and, consequently, affect cell viability, transformation, and malignant progression [95,96].

The effect of Cd on alterations in transcript expression in the HHG axis in males is not fully understood, highlighting the need to understand the molecular mechanisms altered during the onset of puberty upon exposure to this metal. In our study, we observed a temporal pattern of transcriptional alteration in the hypothalamus, characterized by a decrease in *Kiss1* at PND 35, followed by a decrease in *Kiss1r* and *Gnrh1* at PND 49. This suggests a progressive impact of Cd on the kisspeptin-GnRH network, which is essential for the activation and maintenance of the HHG axis in male rats. It has been reported that hypothalamic neurons expressing *Kiss1*, neurokinin B, and dynorphin are crucial for pubertal activation and pulsatile GnRH secretion [10,97,98]. Thus, the early reduction of *Kiss1* at PND 35 and the late reductions of *Kiss1r* and *Gnrh1* at PND 49 are consistent with Cd interfering with GnRH release at the onset of puberty. Cd may interfere with the expression of hypothalamic genes (*Kiss1* and *Gnrh1*), which compromises the neuroendocrine signaling that controls puberty and gonadal hormone production in females [41]. This is consistent with the over-expression of *Kiss1* and the under-expression of *Kiss1r* observed in a 30-day CdCl_2_ exposure in drinking water model in female rats [99]. Similarly, the joint under expression of *Kiss1r* and *Gnrh1* observed in our study is consistent with that seen in a cell model of human fetal neuroblasts exposed to cadmium acetate. This exposure was accompanied by increased oxidative stress, activation of inflammatory pathways, and reduced cell migration and motility. These changes alter the proper maturation of the cells, which could lead to the development of fertility problems in the future [81]. Additionally, decreases in gonadotropins and GnRH pulses associated with Cd exposure have been reported in animal models [31,32,81].

The present study suggests a toxic mechanism by which Cd exposure affects the relative expression of genes involved in hormonal regulation and impacts male pubertal development. It is possible that Cd enters the intracellular environment of hypothalamic neurons through ionic mimicry via voltage-dependent Ca^2+^ channels, thereby mediating Cd toxicity [89,100]. Once inside the neurons, metallothioneins act as the first line of defense by binding to Cd and limiting its neurotoxic effects [101,102]. Additionally, Cd can bind to GSH to form Cd–GSH complexes, which contribute to oxidative stress and redox imbalance [103]. This possibility is supported by the reduction in TAC observed in both serum and hypothalamic tissue, suggesting the occurrence of oxidative stress induced by Cd exposure [36]. Oxidative stress in hypothalamic neurons may compromise signaling pathways that regulate reproductive neuroendocrine function. Furthermore, Cd interferes with Ca^2+^ signaling pathways by competing with Ca^2+^ and binding to calmodulin. This interaction disrupts physiological and biochemical processes regulated by calmodulin, including the induction of transcription factors [100,104]. These alterations may lead to genotoxic effects by inhibiting DNA repair mechanisms [92,105] and dysregulating gene expression [106]. Additionally, Cd exposure may induce epigenetic modifications, such as DNA methylation and histone modifications, resulting in long-term alterations in gene expression patterns [84,106].

Importantly, our results indicate that Cd exposure alters the expression of *Kiss1*, *Kiss1r*, and *Gnrh1* in the hypothalamus, suggesting disruption of the neuroendocrine regulatory network that controls pubertal onset. *Kiss1*-expressing neurons regulate GnRH neurons by activating Kiss1r, thereby stimulating GnRH secretion. This process is essential for the activation of the HHG axis [1,2,7,107]. Therefore, alterations in the expression of these genes may impair GnRH signaling and consequently affect downstream hormonal responses, with significant implications for male pubertal development.

## 5. Strengths and Limitations

This study examined how Cd exposure affects the expression of genes that play a critical role in the onset of puberty and testicular parameters in male rats. Cd exposure altered the expression of genes associated with the *GnRH1* and *Kiss1*/*Kiss1r* signaling systems, delayed pubertal onset, and reduced hypothalamic TAC. These results suggest that the pubertal developmental period is particularly vulnerable to Cd exposure. A strength of this study is its integrative evaluation of molecular, biochemical, and physiological parameters related to reproductive development. This multidisciplinary approach enabled the identification of potential mechanistic links between Cd exposure, oxidative stress, and neuroendocrine alterations affecting the hypothalamic–pituitary–gonadal axis during pubertal development. However, several limitations should be considered. First, a histomorphological analysis of the hypothalamus and the immunolocalization of GnRH neurons were not performed, which could have provided additional information regarding potential structural alterations induced by Cd exposure. Second, circulating levels of the gonadotropins LH and FSH were not measured. Assessing these hormones would have helped further characterize the functional status of the hypothalamic–pituitary–gonadal axis. Third, some methodological parameters recommended by the MIQE guidelines for quantitative PCR analysis were not included. Despite these limitations, the findings of the present study provide valuable information regarding the potential neuroendocrine mechanisms underlying Cd-induced reproductive toxicity. They also contribute to our understanding of how environmental exposure to Cd may affect male pubertal development. Furthermore, these limitations may generate new hypotheses and guide future studies aimed at clarifying the molecular and endocrine pathways involved in Cd-induced neuroendocrine disruption.

## 6. Conclusions

Exposure to Cd during the peripubertal period induces significant alterations in the expression of *Kiss1*, *Kiss1r*, and *Gnrh1* genes, key regulators of pubertal onset. These findings demonstrate that Cd acts as both an endocrine disruptor and neurotoxic agent during a critical reproductive postnatal developmental stage, with potential long-term implications for male reproductive maturity and neuroendocrine regulation.

## Figures and Tables

**Table 1:** TaqMan gene expression assays were used for RT-qPCR analysis.

Gene	TaqMan Assay ID	Amplicon Length (bp)
*Kiss1*	Rn00710914_m1	65
*Kiss1r*	Rn00576940_m1	82
*Gnrh1*	Rn00562754_m1	129
*Gapdh*	Rn01775763_g1	174

Note: TaqMan^®^ Gene Expression Assays were obtained from Thermo Fisher Scientific (Applied Biosystems™, Foster City, CA, USA). *Gapdh* was used as the endogenous reference gene for normalization of gene expression levels.

**Table 2:** Effects of Cd exposure on body and testicular morphometric parameters in Wistar rats during pubertal development.

	PND 35		PND 49	
	Ctrl	Cd	Ctrl	Cd
Body weight (g)	142 ± 0.83	139 ± 0.85 ^a^	230 ± 2.1	220 ± 4.1 ^a^
Body length (cm)	31 ± 0.17	30 ± 0.11 ^a^	39 ± 0.25	38 ± 0.33 ^a^
Testicular weight (g)	0.59 ± 0.015	0.55 ± 0.006	1.2 ± 0.021	0.51 ± 0.019 ^a^
Testicular volume (cm^3^)	0.82 ± 0.005	0.78 ± 0.037	1.7 ± 0.013	0.73 ± 0.060 ^a^
Gonadosomatic index	0.42 ± 0.012	0.40 ± 0.005	0.54 ± 0.008	0.23 ± 0.011 ^a^

Data are expressed as mean ± SEM for all analyzed groups (*n* = 6 per group). Significant differences between Ctrl and Cd-treated groups at the same age are indicated by ^a^ (*p* < 0.05).

**Table 3:** Effects of Cd exposure on serum testosterone levels, Cd accumulation, and total antioxidant capacity in Wistar rats during pubertal development.

	PND 35		PND 49	
	Ctrl	Cd	Ctrl	Cd
Testosterone (ng/mL)	0.53 ± 0.050	0.32 ± 0.035 ^a^	2.20 ± 0.16	0.77 ± 0.13 ^a^
Cd in hypothalamus (µg/g)	0.0001 ± 0.0	0.21 ± 0.039 ^a^	0.0001 ± 0.0	0.34 ± 0.025 ^a^
Cd in blood (µg/mL)	0.001 ± 0.0	0.255 ± 0.018 ^a^	0.0009 ± 0.0	0.354 ± 0.015 ^a^
TAC in hypothalamus (µM)	1.2 ± 0.13	0.50 ± 0.074 ^a^	0.48 ± 0.062	0.27 ± 0.061 ^a^
TAC in serum (µM)	1.74 ± 0.06	1.26 ± 0.13 ^a^	1.67 ± 0.12	1.11 ± 0.02 ^a^

Data are expressed as mean ± SEM for all analyzed groups (*n* = 6 per group). Significant differences between Ctrl and Cd-treated groups of the same age are indicated by ^a^ (*p* < 0.05).

## Data Availability

The original contributions presented in this study are included in the article. Further inquiries can be directed to the corresponding author.

## Abbreviations

The following abbreviations are used in this manuscript:

AAS	Atomic Absorption Spectrophotometer
ARC	Arcuate Nucleus
BBB	The blood–brain barrier
Ca	Calcium
Cd	Cadmium
CdCl_2_	Cadmium Chloride
DNA	Deoxyribonucleic acid
ELISA	Enzyme-Linked Immunosorbent Assay
FSH	Follicle-Stimulating Hormone
*Fshb*	Follicle-Stimulating Hormone Beta subunit
*Gnrh1*	Gen encodes a gonadotropin-releasing hormone
GnRH	Gonadotropin-releasing hormone
GSH	Glutathione
HHG	Hypothalamic–Hypophysis–Gonadal
IP	Intraperitoneal
KISS1	Kisspeptine
*Kiss1*	Gen encodes a protein called kisspeptin
*Kiss1r*	Gen encodes a receptor for kisspeptins
KNDy	KISS1, neurokinin B, and dynorphin A
LH	Luteinizing Hormone
*Lhb*	Luteinizing Hormone Beta Subunit
ME	The Median Eminence
PND	Postnatal Day
PS	Preputial Separation
RT-qPCR	real-time quantitative polymerase chain reaction
-SH	Sulfhydryl group
*Star*	Steroidogenic acute regulatory protein
TAC	Total Antioxidant Capacity
